# Gender Differences in Cardiovascular Pharmacotherapy—the Example of Hypertension: A Mini Review

**DOI:** 10.3389/fphar.2020.00564

**Published:** 2020-05-06

**Authors:** Jacklean Kalibala, Antoinette Pechère-Bertschi, Jules Desmeules

**Affiliations:** ^1^Faculty of Medicine, Geneva University, Geneva, Switzerland; ^2^Division of Nephrology and Hypertension, Geneva University Hospitals, University of Geneva, Geneva, Switzerland; ^3^Division of Clinical Pharmacology and Toxicology, Department of Anesthesiology Pharmacology Emergency Medicine and Intensive Care, Geneva University Hospitals, University of Geneva, Geneva, Switzerland

**Keywords:** hypertension, pharmacology, gender, sex, pharmacokinetics, cardiovascular drugs

## Abstract

Cardiovascular disease (CVD) is the leading cause of mortality worldwide in both sexes. Despite considerable progress in better understanding the patterns of disease in women, they are still often undertreated and benefit less from evidence-based treatment. Hypertension is a key contributor to CVD and is also one of the most potent risk factors for heart failure in women. Even with the wide variety of available drugs, blood pressure control is globally suboptimal. Current guidelines do not suggest differential treatment of hypertension for women; however, a growing body of research suggests gender dimorphism in the pathophysiology of hypertension and pharmacological response to cardiovascular drugs. The clinical relevance of theses sex-divergent effects of drugs is still under investigation. Owing to the exponential relationship between blood pressure and cardiovascular mortality, even a modest decrease in blood pressure or therapeutic adhesion could be clinically \relevant. In this review, we explore the known pharmacological and pharmacokinetic sex differences with special attention to the main classes of antihypertensive treatment. Current data shows frequently higher drug exposures in women and more frequent adverse drug reactions in all antihypertensive drug groups. As far as cardiovascular prevention is concerned, sex-specific data is often lacking in clinical trials, highlighting the necessity to further study CVD and their treatment in both men and women.

## Introduction

For a long time, the risk of cardiovascular disease (CVD) was underestimated in women (Tamargo et al., 2017). While women of childbearing age have a lower risk of cardiovascular (CV) events, this protection decreases after menopause. Although considerable progress has been made in better understanding the patterns of CVD in women, they are still often undertreated, have inadequate prevention and are in turn more vulnerable to CVD.

The World Health Organization (WHO) states that CVD is the leading cause of mortality worldwide in both sexes. An estimated 17.7 million people died from CVDs in 2015 (World Health Organization (WHO), 2017). Hypertension is a key contributor to a global epidemic of CVD that is manifested *via* a range of complications such as stroke, acute coronary syndromes, chronic heart failure and chronic kidney disease. The most potent risk factors for heart failure in women are hypertension and diabetes. For men on the other hand, the main risk factor is coronary heart disease (Regitz-Zagrosek and Seeland, 2013). Epidemiological studies also show that women die more frequently from CVD than men (Regitz-Zagrosek and Seeland, 2013). There is evidence of gender differences not only in the pathophysiology but also in the management and treatment of hypertension (Cadeddu et al., 2016). Furthermore, many studies highlight sex-differences in the pharmacokinetics (PK) of CV drugs.

In this paper we review the known sexually dimorphic pharmacologic and more specifically pharmacokinetic differences. Our review focuses on differences in the main classes of antihypertensive drugs. Our aim is to discuss their clinical relevance, possible impact in explaining the gender discrepancies in the morbidity and mortality of hypertension and to identify scientific gaps.

## General PK

PK describes the process that drugs and other substances undergo in the body. The processes by which the body handles drugs are absorption, distribution, metabolism and elimination. Gender differences in PK have been described in various studies since the 1970s. However, the clinical implications of these differences are still debated. Data in this regard are scarce and sex-specific evidence-based guidelines are rare.

Regarding absorption, compared to men, women have a higher gastric pH, slower gastric emptying and a longer total gastrointestinal (GI) transit time (Freire and Basit, 2011). These differences can affect the bioavailability of drugs requiring an acidic environment for absorption and modified release formulations. Sex-differences in gastrointestinal Glutathione-S-transferase and cytochrome P450 enzymes have been noted, although the number of subjects in these studies is generally too low to draw meaningful conclusions. Several studies have found that men have higher levels of drug efflux pump glycoprotein P (P-gp) in the ileum (Anderson, 2008; Bebawy and Chetty, 2009; Regitz-Zagrosek and Seeland, 2013). However, a recent murine study performed a scan of the whole intestine and found varying levels of P-gp throughout without any sex differences (Bebawy and Chetty, 2009).

Drug distribution is dependent on multiple factors. Women generally weigh less than men and have a higher percentage of body fat. Thus, lipophilic substances will have a higher volume of distribution (Vd) in women and hydrophilic substances a higher Vd in men. Women also have a lower plasma volume and lower average organ blood flow. These differences contribute to divergence in drug distribution and could be considered in order to avoid unnecessary adverse reactions or optimize efficacy. Drug binding proteins are albumin, alpha1-acid-glycoprotein (AAG) and globulins. Albumin does not appear to be sex dimorphic, but estrogens reduce the plasma levels of AAG by inducing its hepatic glycosylation (Parekh, 2012; Spoletini et al., 2012).

There is a large body of literature regarding variability in drug metabolism due to the influence of intrinsic and extrinsic factors. **Table 1** summarizes sex differences in ghepatoc metabolism. Phase I reactions are mainly catalyzed by the cytochrome P450 family of enzymes (CYP). Men have been consistently noted to have higher activities of CYP 1A2 and CYP 2E1 (Anderson, 2005; Anderson, 2008; Parekh, 2012; Regitz-Zagrosek and Seeland, 2013). For CYP 2D6, some studies found higher activities in men, while others found no differences (Anderson, 2005; Anderson, 2008). Women, on the other hand, exhibit higher activities of CYP 3A4 and 2B6 (Anderson, 2005). CYP 3A4 is involved in the metabolism of about 50% of drugs and women frequently have higher clearance of drugs metabolized by this enzyme (Bebawy and Chetty, 2009; Spoletini et al., 2012).There are no consistent sex-differences described for CYP 2C19 and there are several studies that do not find any sex differences in the clearance of drugs metabolized by CYP 2C9 (Hägg et al., 2001; Anderson, 2005). There is evidence of some hormonal regulation of hepatic drug metabolism. Estrogen and hormonal contraception reduce hepatic blood flow and induce CYP 2A6 and 2B6 (Parekh, 2012). CYP 2C19 activity was shown to be 60% lower in women receiving oral contraceptives (Hägg et al., 2001; Parekh, 2012; Spoletini et al., 2012). P-gp and CYP 3A4 also seem to be subject to hormonal regulation (Bebawy and Chetty, 2009).

**Table 1:** Sex differences in hepatic metabolism and transporters (P-Gp).

	Sex difference	Hormonal influence
**CYP 1A2**	Female > Male	Inhibited by oral contraceptives
**CYP2A1**	Female < Male	
**CYP 2A6**	Female > Male	Induced by estrogens and oral contraceptives
**CYP 2B6**	Female > Male	Induced by estrogens and oral contraceptives
**CYP 2E1**	Female < Male	
**CYP2D6**	Conflicting data	
**CYP 2C9**	Female = Male	
**CYP 2C19**	No consistent data	Inhibited by oral contraceptives
**CYP 3A4**	Female > Male	Induced by testosterone and progesterone.
**UGTs**	Female < Male	Induced by oral contraceptives
**NATs**	Female = Male	
**TPMT**	Female < Male	
**P-Gp**	Female < Male	Induced by estrogens Inhibited by testosterone

CYP, cytochrome P450; NATs, arylamine N-acetyltransferase; UGTs, Uridine 5′-diphospho-glucuronosyltransferases; TPMT, Thiopurine methyl transferase; P-Gp, glycoprotein P.

Regarding phase II metabolism, UGT-conjugation (or glucuronidation) of some drugs (oxazepam, paracetamol) has been reported to have faster clearance in men. A study on paracetamol clearance in young women found that oral contraceptives increased paracetamol glucuronidation clearance by a factor of 1.46 compared to women without oral contraception (Allegaert et al., 2015). There was also an increase of glucuronidation in women at delivery (factor 2.03), and a decrease in the early postpartum (factor 0.55). However, these differences are not consistent, there were for example no differences noted for ibuprofen (Tamargo et al., 2017). To date, there are no consistent sex-differences reported for acetylation or sulfation (Regitz-Zagrosek and Seeland, 2013; Tamargo et al., 2017). Thiopurine methyl transferase (TPMT) activity is subject to genetic polymorphism and higher levels or TPMT have been reported in men. A Spanish study on 14,545 patients found a small but statistically significant influence of gender on TPMT activity whereas male patients had a slightly higher activity (male, 20.4 U/ml; female, 19.9 U/ml p < 0.001) (Gisbert et al., 2007).

In terms of excretion, glomerular filtration rate, tubular secretion, and reabsorption are important determinants. Studies have shown that women have a GFR of 10%–25% lower than men even after adjusting for body size (Anderson, 2005; Parekh, 2012). In regards to tubular expression, animal models have shown differences between males and females throughout the nephron. Females show lower sodium and water reabsorption in proximal nephron but a greater abundance of key transporters in the distal nephron (Veiras et al., 2017; Li et al., 2018; Hu et al., 2019).

## Treatment of Hypertension

### Women and Hypertension

As mentioned above gender disparity in the epidemiology of hypertension is evident. Young women have lower systolic blood pressure (SBP) compared to age-matched men, but the prevalence of hypertension in women is higher in postmenopausal women reaching 78% above the age of 75 in the United States (Whelton et al., 2018). Furthermore, there is evidence of gender dimorphism in not only the epidemiology but also the pathophysiology of hypertension (Cadeddu et al., 2016). Disparities may be related to biological factors as well as differences in health care access and response to therapy. Current data is insufficient to support differential treatment for women. Recent European society of hypertension and cardiology (ESH and ESC) guidelines (2018) and American Heart Association (AHA) guidelines (2017) on hypertension recommend the same blood pressure (BP) targets and treatments for both sexes (Whelton et al., 2018; Williams et al., 2018). Despite these guidelines, there is evidence of gender dimorphic management and control, for example BP control seems to be poorer in young men and older women (Thoenes et al., 2010; Sarganas and Neuhauser, 2016).

Several studies have noted differences in the choice of antihypertensive drug classes. Men are more often prescribed angiotensin converting enzyme inhibitors (ACEI), angiotensin II receptor blockers (ARB), and beta blockers whereas women generally receive diuretics and calcium channel blockers (CCB) (Thoenes et al., 2010; Cadeddu et al., 2016). One of the reasons why young women are less often prescribed ACEIs and ARBs is the risk of pregnancy. Physicians have to take this contraindication into consideration and discuss the risk or choose a safer drug class (Suchard et al., 2019).

In addition, there appears to be a gender dimorphism in salt-sensitive hypertension. After menopause women have been noted to become more sodium sensitive, suggesting a hormonal influence in salt handling (Pechère-Bertschi and Burnier, 2004; Doumas et al., 2013; Cadeddu et al., 2016). This theory is supported by the reported benefits of salt restriction in older women and experimental animal models. Studies on salt sensitive rats, have shown a lower proportion of development of hypertension in female rats compared to male rats when put on a high-sodium diet (Pechère-Bertschi and Burnier, 2004; Cadeddu et al., 2016). Furthermore, in these same female rats, ovariectomy resulted in increased salt-sensitive hypertension.

### Pharmacological Treatment and CV Prevention

Despite the large number of clinical trials for the treatment of hypertension, specific data for the women is not easily obtained because in many of these studies women are underrepresented or a gender specific analysis is not performed. In terms of beneficial effects, a meta-analysis examined the effects of BP lowering treatments in men and women and found no significant sex-differences in CV outcomes for all drug classes except for stroke (Turnbull et al., 2008). For stroke prevention in women CCBs seemed superior to ACEIs (p = 0.05).

The presence of even minor sex-differences in some studies raises the question of the generalization of their results. For example, the SPRINT trial which generated a change in the recent AHA guidelines, after comparing intensive SBP targets (< 120 mmHg) and standard targets (< 140 mmHg), only included 36% women (SPRINT Research Group et al., 2015). The authors found an overall reduction in the primary outcome of CV events for the intensive group (p < 0.001). They found no significant gender-treatment interaction (p= 0.45). The hazard ratio (HR) for the women subgroup was 0.84 with a 95% CI between 0.62-1.14 suggesting that the difference was not significant. Another example is the Accomplish trial which compared a combination of an ACEI +CB to ACEI +hydrochlorothiazide in high risk patients (Jamerson et al., 2008). The results showed superiority of the ACEI + CCB combination in reducing CV events and death from CV events (p < 0.001). The study included 39.5% women and the results did not reach significance for this subgroup (p = 0.06).

**Table 2** shows a summary of the key hypertension trials of the last 2 decades that included sex-specific analysis. Overall, the studies show no significant gender treatment interaction but on closer examination some differences are noteworthy and deserve further investigation. The details of specific PK sex-differences for each drug class will be reviewed in the following sections and summarized in **Table 3**.

**Table 2:** Summary of landmark clinical trials for hypertension.

Trial	No. of patients: Women Men	Study population	Treatment arms	Primary outcome	Results	Sex-specific analysis	ref
**LIFE** **(2002)**	n=9,193 4,963 4,230	Adults 55 to 89 years with hypertension and LVH	Losartan vs atenolol	Composite CV event (death, MI or stroke)	Losartan superior to atenolol for primary outcome and stroke outcome	–Not tested for primary outcome. –stroke outcome: no sex-differences (p = 0.47). –Regression of left ventricular mass lower in women	(Kizer et al., 2005; Okin et al., 2008)
**ALL-HAT** **(2003)**	n=3,3357 15,638 17,719	Adults >55 years with hypertension and at least 1 other risk factor for CHD	Chlortalidone vs amlodipine/lisinopril	Fatal CHD or nonfatal MI	No differences in primary outcome between treatment groups	–No sex differences in primary outcome –Stroke outcome: stroke rate significantly higher for women with ACEI compared to chlorthalidone (HR 1.21) and amlodipine (HR 1.45)	(Leenen et al., 2006; Oparil et al., 2013)
**ANBP2** **(2003)**	n=6,083 3,102 2,981	Adults 65-84 years with hypertension	Enalapril vs hydrochlorothiazide	Composite of Major CV event or death	ACEI superior to THZ for primary outcome	–Primary outcome: No difference between treatment groups for women (HR 1.00) –Half as many events in women	(Wing et al., 2003)
**Value** **(2006)**	n=15,245 6,468 8,777	Adults at high risk of hypertension	Valsartan vs amlodipine	CV morbidity and mortality	No significant difference in primary outcome between treatment groups	–Primary outcome: Amlodipine superior for women (p= 0.02) –HF outcome: valsartan superior for men (p < 0.001)	(Zanchetti et al., 2006)
**Accomplish** **(2008)**	n=11,506 4,542 6,964	Adults with systolic hypertension	Benazepril + amlodipine vs benazepril + hydrochlorothiazide	Composite of CV event and death from CV event	CCB + ACEI superior for primary outcome	–For women subgroup: difference between treatment groups not significant for primary outcome (p= 0.06)	(Jamerson et al., 2008)
**HYVET** **(2008)**	n= 3,845 2,326 1,519	Elderly > 80 years	Indapamide +/− perindopril vs placebo	Any stroke (fatal or nonfatal)	Indapamide with or without ACEI is associated with reduction in stroke (p = 0.06) and death from any cause (p = 0.02)	-No reporting of sex-specific data –large female subgroup (60%)	(Beckett et al., 2008)
**SPRINT (2015)**	n=9,361 3,332 6,029	Adults >50 years with hypertension and increased CV risk	SBP target <130 mmHg (intensive) vs <140 mmHg (standard)	Composite of MI, acute CHD, stroke, HF or death from CV causes	Intensive treatment superior to standard for primary outcome	–No sex-differences (p= 0.45) –For women subgroup: HR not significant (HR 0.84, 95% CI 0.62–1.14	(SPRINT Research Group et al., 2015)
**Hygia (2019)**	n=19,084 8,474 10,610	Adults > 18 years with hypertension	Bedtime treatment vs awakening	Major CV event (CVD death, MI, coronary revascularization, HF or stroke)	Bedtime treatment superior to awakening for primary outcome	No sex-differences	(Hermida et al., 2019)

LVH, left ventricular hypertrophy; CV, cardiovascular, MI, myocardial infarction; CHD, coronary heart disease; HF, heart failure; ACEI, angiotensin converting enzyme inhibitors; CCB, calcium channel blockers, THZ, Thiazide diuretic; HR, hazard ratio.

**Table 3:** Pharmacological sex-differences in antihypertensive treatment.

Drug/Drug class	Sex difference	References
**ARBs**	No PK sex-differences for candesartan, losartan and valsartan (after adjustment for weight). Higher Cmax in women for telmisartan compared to men.	(Rabi et al., 2008; Thoenes et al., 2010; Cabaleiro et al., 2013)
**ACEIs**	Enalapril: –No PK sex-differences –Lower minimum ACE activity after administration of enalapril in women compared to men. More frequent cough in women compared to men.	(Zapater et al., 2004; Rabi et al., 2008; Thoenes et al., 2010)
**Beta-blockers**	Metoprolol: –lower oral Cl in women compared to men. –50% higher Cmax and AUC in women compared to men. More adverse drug reactions for CYP 2D6 dependent beta-blockers (metoprolol, carvedilol, nebivolol and propranolol) in women. Women are less sensitive to sympathetic vasoconstrictor activity compared to men.	(Luzier et al., 1999; Thürmann et al., 2006; Eugene, 2016)
**Calcium channel blockers**	Amlodipine: –No PK sex-differences (after adjustment for weight). –Greater changes in BP for women compared to men. Verapamil: –Higher IV Cl in women compared to men. –Lower oral Cl in women compared to men. Higher incidence of edema in women compared to men.	(Kloner et al., 1996; Krecic-Shepard et al., 2000; Abad-Santos et al., 2005; Dadashzadeh et al., 2006)
**Thiazide diuretics**	Hydrochlorothiazide: –No PK sex-differences More frequent hyponatremia and hypokalemia in women compared to men.	(Musini et al., 2014; Rydberg et al., 2018)

ARB, Angiotensin receptor blocker; ACEI, Angiotensin converting enzyme inhibitor; Cmax, peak concentration; AUC, area under the curve; PK, pharmacokinetic; Cl, clearance; ADR, adverse drug reactions.

### Drugs That Target the Renin-Angiotensin-Aldosterone System

A main determinant in hypertension is the renin-angiotensin-aldosterone system (RAAS). The activity of the RAAS is higher in men than in premenopausal women (Komukai et al., 2010; Doumas et al., 2013). There is evidence of hormonal regulation of the RAAS which could explain the postmenopausal rise in BP. In postmenopausal women, angiotensinogen levels are increased by oral estradiol administration but not by percutaneous administration, which could suggest an effect on hepatic angiotensinogen synthesis (Komukai et al., 2010). Estrogens also inhibit the sympathetic nerve discharge which in turn stimulates renin production. This suggests that the effects of estrogens on the RAAS might also be mediated by their effects on the sympathetic activity (Komukai et al., 2010; Cadeddu et al., 2016).

In experimental models, androgens stimulate the RAAS whereas estrogens and progesterone reduce plasma renin activity, angiotensin converting enzyme (ACE) activity, and aldosterone levels (Pechère-Bertschi and Burnier, 2004; Komukai et al., 2010). Studies on female rats have shown that ovariectomy increases blood pressure and this effect is reversed by administration of subcutaneous estrogen (Komukai et al., 2010). There is also evidence of sex-differences in angiotensin receptor expression; in animal models AT1 receptor expression is lower in the renal cortex of female rats, and ovariectomy increases receptor density (Komukai et al., 2010). AT2 receptor stimulation is believed to antagonize AT1. Evidence from murine studies suggest that AT2 receptor expression and binding are increased by estrogen (Komukai et al., 2010).

Drugs that target the RAAS are the most prescribed antihypertensive drugs (Rabi et al., 2008; Thoenes et al., 2010); they have comparable effects on blood pressure lowering in both sexes. A literature review (Rabi et al., 2008) of controlled trials of ACEI and angiotensin-receptor blockers (ARB) found that sex specific data was only available for 43% of the studies, with only six ACEI studies and three ARB studies reporting sex-specific outcomes (Rabi et al., 2008). Although some Clinical trials show minor sex-differences in CV prevention (Value, ANBP2) the overall reporting of sex-specific data is insufficient.

A bioequivalence study of enalapril formulations administered to healthy volunteers found no significant pharmacokinetic sex-differences (Zapater et al., 2004). However, pharmacodynamic gender differences were observed. Firstly, Women had lower minimum ACE activity after administration of enalapril. Second, the inhibition of ACE activity in women was dependent on the plasma enalaprilat (active metabolite) concentration. For concentrations <5 ng/ml, plasma ACE inhibition was lower in women. Over 5 ng/ml the same level of ACE inhibition was obtained for men and women. Lastly, the authors noted lower SBP and ACE activity in women at all enalaprilat concentrations even at maximum inhibition of ACE activity.

Calbaleiro et al. (2013) evaluated sex-differences in the PK of commonly used ARBs (candesartan, telmisartan, losartan, and valsartan) and found overall higher Cmax (concentration) and AUC (area under the curve) in women. These differences were not significant when adjusted to weight. The only difference that remained significant was a higher Cmax for telmisartan without significant differences in BP changes. For the authors this sex dimorphism is due to slower clearance in women as reported with other drugs that are metabolized by conjugation or oxidation.

### Thiazide Diuretics

Thiazide diuretics (TD) are commonly prescribed as monotherapy for treatment of hypertension. A Cochrane review on their efficacy as monotherapy showed a dose related BP lowering response to hydrochlorothiazide with a greater effect on SBP. Due to insufficient data reporting of specific patient characteristics subgroup analysis for sex could not be carried out (Musini et al., 2014). LEGEND-HTN, a recent large-scale meta-analysis (4.9 million patients) comparing first-line antihypertensives on patients initiating treatment found that patients on TD showed lower risks of all primary outcomes (Myocardial infarction, hospitalization for HF and stroke) and mortality compared to ACEI and CCB. Only 17% of the patients were prescribed TD compared to 48% prescribed ACEI. However, women were more often prescribed TD so the greater benefits of TDs could be due to more beneficial effects on women (Suchard et al., 2019). Indeed, TDs are often used in women; this may be because of their beneficial effect on decreased renal excretion of calcium, bone density and reduction of fracture risk in postmenopausal women (Thoenes et al., 2010). Numerous studies have shown that female sex is a risk factor for hyponatremia in TD users (Hwang and Kim, 2010; Rodenburg et al., 2010). Data from a recent Swedish study on spontaneously reported adverse drug reactions (ADR) for common antihypertensive drugs found that 86% of the reports for TD induced hyponatremia were for women (Rydberg et al., 2018). Another study found that women were five times more likely to be hospitalized for hyponatremia due to diuretics (Cadeddu et al., 2016). Surprisingly, one population-based study showed conflicting results; women, who represented 59% of the study population, did have a higher but not significant risk or hyponatremia compared to men. BMI and younger age were however significant risk factors (Rodenburg et al., 2013).

Pharmacogenetic factors are important determinants in pharmacological response to therapy. There may be an interaction between sex and genetics. Schwartz et al. (2002) suggest a gender specific interaction between insertion/deletion polymorphism in the angiotensin I-converting enzyme (ACE) gene and the response to hydrochlorothiazide (HCTZ). In their study the genotype predicted BP response to HCTZ in a gender specific manner. Women with more I alleles had increasingly better BP response whereas the BP response in men increased with the number of D alleles (Schwartz et al., 2002). The gender-genotype relationship was an independent factor for BP response even after adjustment for race, age and body size. However, ACE activity increased in association with D alleles in both sexes suggesting that the effects of the I/D polymorphism on ACE activity alone cannot explain this phenomenon.

### Beta-Blockers

The CV system is controlled and regulated by the noradrenergic system. Premenopausal women have lower sympathetic nerve activity than age matched men and also appear to be less sensitive to sympathetic vasoconstrictor activity (Franconi and Campesi, 2014).

Although beta-blockers have been found to be inferior to other drug classes in terms of CV prevention (Ettehad et al., 2016), they are still proposed as secondary agents for patients with certain co-morbidities such as heart failure and ischemic heart disease (Whelton et al., 2018; Williams et al., 2018). There are important pharmacokinetic gender-differences in the disposition of β-antagonist metoprolol. One study on healthy volunteers found significantly lower oral clearance in women (Luzier et al., 1999). Gender differences were also found in Cmax, and AUC which were both greater in women. Differences of nearly 50% persisted even after normalization for body weight. The study found no differences in terminal half-life. These differences may be explained by differences in body composition or in bioavailability. There were also differences in pharmacodynamic response to metoprolol. Women had greater reduction in heart rate and BP, but these differences disappeared when adjusted for serum concentration. In line with these reports a pharmacokinetic simulation and modeling study suggests a 50% dose reduction of metoprolol in healthy young women compared to men (Eugene, 2016).

Cytochrome 2D6 is known to have a genetic polymorphism. A population based retrospective study of adverse effects of β-blockers in Germany found a fivefold higher frequency of CYP 2D6 poor metabolizers in patients with adverse effects (Wuttke et al., 2002). Notably, in this cohort of patients with adverse events, the proportion of CYP 2D6 poor metabolizers was three times higher than that of the general German population and 75% of the patients were women. Secondary analysis of data from a longitudinal cohort study on hospital admissions caused by adverse drug events in Germany examined the interaction between gender and adverse effects of beta-blockers (Thürmann et al., 2006). B-blockers were divided into two groups: CYP 2D6-dependant (metoprolol, carvedilol, nebivolol, and propranolol) and CYP2D6-independent (sotalol, bisoprolol, and atenolol). The authors found that the number of ADR associated with CYP 2D6-dependant β-blockers was significantly higher in women than in men. But for CYP 2D6-independent β-blockers there was no gender disparity. Interestingly drug interaction with other heart rate lowering drugs was also more frequent in women with CYP 2D6-dependant β-blockers (Thürmann et al., 2006).

### Calcium Channel Blockers

Pharmacokinetic sex-differences have been noted for some calcium channel blockers. For amlodipine, a long acting dihydropyridine calcium channel blocker, bioavailability was found to be higher in women (AUC and Cmax), but not when adjusted for weight (Abad-Santos et al., 2005). Pharmacodynamic differences have also been noted; one multicentric study with 1,000 patients found greater BP changes from baseline in women as well as a higher percentage of women achieving BP target goal (Kloner et al., 1996). These differences in changes in BP remained significant event after adjusting for baseline characteristics like weight, age, and dose/kg. Women were also found to have a significantly higher incidence of edema (Kloner et al., 1996). Regarding CV prevention, CCBs seem to be superior to other drug classes for stroke prevention (Ettehad et al., 2016) with some studies showing greater benefit for women (Zanchetti et al., 2006; Oparil et al., 2013).

Verapamil another calcium channel blocker showed faster IV clearance in women compared to men (Krecic-Shepard et al., 2000). Another study also reported faster oral clearance of verapamil and a shorter half-life in healthy female volunteers compared to their male counterparts (Dadashzadeh et al., 2006). Verapamil undergoes extensive first pass hepatic metabolism and is a substrate of both CYP 3A4 and P-glycoprotein. Hence the faster clearance in women could be due to higher CYP 3A4 activity in women or to low P-Gp activity which would result in increased intrahepatic substrate for CYP 3A4 (Krecic-Shepard et al., 2000; Dadashzadeh et al., 2006). Krecic-Shepard et al. (2000) also report slower oral clearance of verapamil in women. Differences between i.v and oral routes could be due to gender differences in intestinal CYP 3A4 and P-Gp activity although no differences in time to peak concentrations were noted to support this theory. As far as pharmacodynamics are concerned, changes in BP after verapamil administration did not differ between men and women (Krecic-Shepard et al., 2000).

## Conclusion

Regarding the treatment of hypertension, an increasing number of studies show interactions between gender and PK as well as pharmacogenetics, that could influence BP control and prevention of CV events. On the whole women seem to be more prone to ADR in all antihypertensive drug groups. Although gender differences in drug toxicity are often attributed to differences in body weight and composition, several studies reviewed here show persistent gender dimorphism even after adjustment for these factors. This suggests that perhaps introduction of antihypertensive treatment in women should be done with closer attention to adverse drug events. Thus, a gender-based selection of pharmacological treatment could lead to better BP control and therapeutic adherence. Furthermore, the frequently higher drug exposure in women raises the question of the necessity of dose reduction in this population, as it has been suggested for other subgroups of patients (i.e. geriatric patients). Dose adjustments could be beneficial not only for purely pharmacokinetic considerations but also for CV prevention; this theory has recently been tested for the treatment of heart failure but remains to be explored for hypertension.

These observations underscore the importance of studying diseases and their treatment in men and women. Several advances have been made in order to reduce the dearth of knowledge and awareness of gender differences in CVDs. Gender differences in pharmacotherapy have been observed in other CVDs such as heart failure and stroke, and in other areas of medicine. The main issue hindering a comprehensive approach seems to be lack of consistent sex-specific data, as noted by several authors. With the advent of personalized medicine there is a common agreement that gender differences in pharmacotherapy should be studied systematically and gender should be included in covariate analyses and not only in *post hoc* analysis.

## Author Contributions

Conceptualization: JK. Writing original draft preparation: JK. Writing review and editing: JK, AP-B, and JD. Supervision: AP-B and JD.

## Conflict of Interest

The authors declare that the research was conducted in the absence of any commercial or financial relationships that could be construed as a potential conflict of interest.
